# A Multi-Center Study of CT-Based Neck Nodal Radiomics for Predicting an Adaptive Radiotherapy Trigger of Ill-Fitted Thermoplastic Masks in Patients with Nasopharyngeal Carcinoma

**DOI:** 10.3390/life12020241

**Published:** 2022-02-06

**Authors:** Sai-Kit Lam, Jiang Zhang, Yuan-Peng Zhang, Bing Li, Rui-Yan Ni, Ta Zhou, Tao Peng, Andy Lai-Yin Cheung, Tin-Ching Chau, Francis Kar-Ho Lee, Celia Wai-Yi Yip, Kwok-Hung Au, Victor Ho-Fun Lee, Amy Tien-Yee Chang, Lawrence Wing-Chi Chan, Jing Cai

**Affiliations:** 1 Department of Health Technology and Informatics, The Hong Kong Polytechnic University, Hong Kong, China; saikit-edmond.lam@connect.polyu.hk (S.-K.L.); jiang.zhang@connect.polyu.hk (J.Z.); y.p.zhang@ieee.org (Y.-P.Z.); zlyylibing4127@zzu.edu.cn (B.L.); raven.nr.ni@connect.polyu.hk (R.-Y.N.); ta.zhou@polyu.edu.hk (T.Z.); Tao.Peng@UTSouthwestern.edu (T.P.); CLY916@ha.org.hk (A.L.-Y.C.); wing.chi.chan@polyu.edu.hk (L.W.-C.C.); 2 Department of Clinical Oncology, Queen Mary Hospital, Hong Kong, China; 3 Department of Clinical Oncology, The University of Hong Kong, Hong Kong, China; chautc@hku.hk (T.-C.C.); vhflee@hku.hk (V.H.-F.L.); 4 Department of Clinical Oncology, Queen Elizabeth Hospital, Hong Kong, China; leekh4@ha.org.hk (F.K.-H.L.); ywy923@ha.org.hk (C.W.-Y.Y.); akhz01@ha.org.hk (K.-H.A.); 5 Comprehensive Oncology Centre, Hong Kong Sanatorium & Hospital, Hong Kong, China; amy.ty.chang@hksh.com

**Keywords:** adaptive radiotherapy, neck lymph node shrinkage, radiomics, thermoplastic mask unfit

## Abstract

Significant lymph node shrinkage is common in patients with nasopharyngeal carcinoma (NPC) throughout radiotherapy (RT) treatment, causing ill-fitted thermoplastic masks (IfTMs). To deal with this, an ad hoc adaptive radiotherapy (ART) may be required to ensure accurate and safe radiation delivery and to maintain treatment efficacy. Presently, the entire procedure for evaluating an eligible ART candidate is time-consuming, resource-demanding, and highly inefficient. In the artificial intelligence paradigm, the pre-treatment identification of NPC patients at risk for IfTMs has become greatly demanding for achieving efficient ART eligibility screening, while no relevant studies have been reported. Hence, we aimed to investigate the capability of computed tomography (CT)-based neck nodal radiomics for predicting IfTM-triggered ART events in NPC patients via a multi-center setting. Contrast-enhanced CT and the clinical data of 124 and 58 NPC patients from Queen Elizabeth Hospital (QEH) and Queen Mary Hospital (QMH), respectively, were retrospectively analyzed. Radiomic (R), clinical (C), and combined (RC) models were developed using the ridge algorithm in the QEH cohort and evaluated in the QMH cohort using the median area under the receiver operating characteristics curve (AUC). Delong’s test was employed for model comparison. Model performance was further assessed on 1000 replicates in both cohorts separately via bootstrapping. The R model yielded the highest “corrected” AUC of 0.784 (BCa 95%CI: 0.673–0.859) and 0.723 (BCa 95%CI: 0.534–0.859) in the QEH and QMH cohort following bootstrapping, respectively. Delong’s test indicated that the R model performed significantly better than the C model in the QMH cohort (*p* < 0.0001), while demonstrating no significant difference compared to the RC model (*p* = 0.5773). To conclude, CT-based neck nodal radiomics was capable of predicting IfTM-triggered ART events in NPC patients in this multi-center study, outperforming the traditional clinical model. The findings of this study provide valuable insights for future study into developing an effective screening strategy for ART eligibility in NPC patients in the long run, ultimately alleviating the workload of clinical practitioners, streamlining ART procedural efficiency in clinics, and achieving personalized RT for NPC patients in the future.

## 1. Introduction

Radiotherapy (RT) is a cornerstone modality for nasopharyngeal cancer (NPC) patients [1,2], among which the involvement of neck lymph nodes (LNs) is of high prevalence [3]. Irradiation down to the cervical LNs, in addition to the primary NPC tumor, is essential for achieving thorough cancer eradication and mitigating the risk of cancer recurrence [4,5]. Throughout the 6–7 weeks of a RT course, a thermoplastic mask (TM) immobilization device that provides full coverage of the head and bi-lateral shoulders is deployed for each NPC patient to ensure reproducible patient positioning between RT fractions in order to maintain treatment efficacy [6]. However, anatomic variations and body weight loss of NPC patients are not uncommon [7,8,9,10,11,12,13], posing a risk of TM unfit. In cases of ill-fitted TMs (IfTMs), an ad hoc adaptive radiotherapy (ART) may be triggered to ensure accuracy and safe radiation delivery and to maintain treatment efficacy [14,15,16,17]. Presently, clinical ART practice is still in its infant stage. The entire procedure for evaluating an eligible ART candidate is time-consuming, resource-demanding, and requires multidisciplinary efforts [7,18]. In the artificial intelligence paradigm, the pre-treatment identification of NPC patients at risk for IfTMs has become greatly demanding for the sake of improving medical resource allocation and achieving greater procedural efficiency in oncologic care delivery.

The volumetric shrinkage of neck nodal lesions is a key factor for IfTM. Since neck nodal lesions locate in a close proximity to the body skin surface of NPC patients, the significant shrinkage of neck LNs in response to treatment would cause a palpable change in the patient’s neck contour, producing a TM-to-skin air gap which in turn elevates the risk of intra-fractional patient movement during RT delivery and hence jeopardizes treatment efficacy [6,19,20,21]. Indeed, there is mounting evidence indicating that substantial LN volume shrinkage occurs throughout the RT course in NPC and head-and-neck cancer (HNC) patients, potentially triggering ART implementation [8,9,22,23,24]. For example, Wing et al. quantified the anatomic changes of 30 NPC patients and reported that there was a significant regression of neck volumes over time with a mean loss rate of 0.39 ± 0.15%/day and a mean volume loss of 11.91 ± 5.57% upon treatment completion [22]. Murat et al. reported that there was a 43% reduction of neck nodal target volumes in HNC patients undergoing RT [23]. Similarly, Cheng et al. reviewed both mid-treatment computed tomographic (CT) and magnetic resonance (MR) scans of NPC patients and showed that there were up to 30% reductions in the volume of nodal lesions [24]. Despite the above evidence, inter-patient heterogeneity in treatment response has impeded the accurate individualized prediction of tumor shrinkage for decades.

Recently, radiomics, which involves the extraction of high-throughput quantitative features from medical images, has become an emerging area for divulging the intrinsic biologic and genetic characteristics of tissue for individual cancer patients [25,26,27,28]. Radiomics has been extensively studied for treatment response prediction in various cancer types on the basis of Response Evaluation Criteria in Solid Tumors (RECIST), where criteria are determined by the extent of tumor shrinkage following treatment [29,30,31,32,33,34,35,36]. For instance, Hou et al. investigated CT-based biomarkers for the prediction of the therapeutic response to chemoradiotherapy in esophageal carcinoma and reported that the discriminability of their model achieved area under the receiver operating characteristics curves (AUC) ranging from 0.686 to 0.727 [29]. Wang et al. developed a radiomic signature combining features from multi-modal MR imaging sequences for the prediction of an early treatment response to induction chemotherapy in NPC patients, achieving an AUC of 0.822 [30]. Piao et al. devised an MR-based radiomic model to distinguish sensitive and resistant tumors in NPC patients following induction chemotherapy, yielding an AUC of 0.905 [31]. These research efforts have laid a great foundation for the radiomics prediction of ART eligibility in cancer patients. Ramella et al. performed a radiomic analysis on pre-treatment CT images of replanned non-small cell lung cancer patients and generated a radiomic signature for the prediction of tumor shrinkage during chemoradiotherapy, yielding an AUC of 0.82 [37]. Yu et al. investigated MR-based radiomics from primary NPC tumors for predicting ART eligibility in a single cohort, achieving AUCs ranging from 0.75 to 0.93 [13].

Unlike MR imaging, CT is often the first-line modality for the neck nodal imaging of NPC patients. In this study, we aimed to investigate the capability of CT-based neck nodal radiomics for predicting IfTM-triggered ART events in NPC patients via a multi-center setting. The main contributions of this study are as follows:

- The application of CT-based neck nodal radiomics for developing a prediction model for IfTM-triggered ART events in NPC patients is proposed for the first time.
- The multi-center setting of this study allows for the assessment of model generalizability across medical institutions.
- The use of radiomics renders the possibility for the pre-treatment identification of NPC patients who are at a greater risk of experiencing IfTM-triggered ART events, potentially alleviating the workload of clinical practitioners, streamlining ART procedural efficiency in clinic, and achieving personalized RT for NPC patients in the future.

The overall structure of this manuscript is organized as follows: Section 2 describes the materials and methods employed in this study; Section 3 presents the findings of this work; and Section 4 and Section 5 refer to the discussion and conclusions, respectively.

## 2. Materials and Methods

### 2.1. Patient Data

A total of 261 NPC patients who received RT at Hong Kong Queen Elizabeth Hospital (QEH) between 2012 and 2015 and 160 NPC patients who received RT at Hong Kong Queen Mary Hospital (QMH) between 2012 and 2020 were retrospectively screened for study eligibility. Patient informed consent was waived due to the retrospective nature of this study. Patients who had biopsy-proven primary NPC without the existence of distant metastasis and co-existing tumors of other types at diagnosis and who received curative concurrent chemoradiotherapy (CCRT) were included in this study. Patients who were treated by induction chemotherapy or did not have a complete set of clinical/image data were excluded from this study. The clinical records of all the enrolled patients were input by the attending radiation oncologists and were carefully examined to determine the binary prediction outcome in this study. Patients who had clinical records regarded as ill-fitted with the TM, necessitating the implementation of ART, were labelled as 1, and were otherwise labeled as 0. 

### 2.2. Image Acquisition and Volume-of-Interest (VOI) Definition

All the planning contrast-enhanced CT (CECT) images were retrospectively collected in the format of Digital Imaging and Communications in Medicine (DICOM) and archived using a picture archiving and communication system (PACs). 

At QEH, intravenous (IV) CECT simulation was performed in a supine position with an immobilization thermoplastic cast. This was typically acquired at 3 mm intervals from the vertex to 5 cm below the sternoclavicular notch under a 16-slice Brilliance Big Bore CT scanner (Philips Medical Systems, Cleveland, OH). CECT acquisition parameters were as follows: scan mode = helical, voltage = 120 kVp, X-ray tube current = 264 mA, exposure = 325 ms, pixel spacing = 1.152 × 1.152-mm, slice thickness = 3 mm, and matrix = 512 × 512 pixels. Two types of IV contrast agents were available: (i) OMNIPAQUE TM 350 mg I/mL and (ii) VISIPAQUE TM 320 mg I/mL; either one of them was prescribed to each eligible patient and was injected at a rate of 2 mL/s for 70 mL, followed by scanning after a 30 s delay.

At QMH, IV CECT simulation was performed in a supine position with an immobilization thermoplastic cast. This was typically acquired at 3 cm intervals from the vertex to 5 cm below the sternoclavicular notch under a 16-slice GE Discovery RT CT scanner (General Electric). CECT acquisition parameters were as follows: scan mode = helical, voltage = 120 kVp, X-ray tube current = 40 mA, exposure = 325 ms, pixel spacing = 1.152 × 1.152 mm, slice thickness = 2.5 mm, and matrix = 512 × 512 pixels. IV contrast agents OMNIPAQUE TM 300 mg I/mL was prescribed to patient and the injection rate was 2 mL/s for 100 mL, followed by scanning after a 20 s delay.

In both hospitals, the gross-tumor-volume of NPC nodal lesions (GTVn) was chosen as the VOI for the extraction of radiomic features in this study, which was manually delineated on axial CT slices by experienced radiation oncologists specializing in head-and-neck cancers with accreditations, using Eclipse ARIA (Varian Medical System, Inc.,Palo Alto, CA, USA) version 13 at QEH and version 13.6 at QMH.

### 2.3. Image Pre-Processing

Given that variations in image acquisition and reconstruction parameters within and between medical centres exist, image pre-processing prior to radiomic feature extraction is of paramount importance for optimizing the consistency and hence reproducibility and validity of radiomics studies. In this study, four key image pre-processing steps were involved, including voxel size resampling, VOI re-segmentation, image filtering, and the quantization of grey levels. All these steps were performed in accordance with well-accepted recommendations from the Image Biomarker Standardisation Initiative (IBSI) guidelines [38], using an in-house developed pipeline tool based on Python v3.7.3. 

First of all, the CECT images were resampled to a voxel size of 1 mm × 1 mm × 1 mm using linear interpolation to correct for different imaging voxel spacing and slice thicknesses among the different institutions. VOI re-segmentation was then performed to confine the Hounsfield unit (HU) to the range of (−150,180) within the VOI for eliminating non-tumor components, such as air cavities and bony structures. Subsequently, Laplacian-of-Gaussian (LoG) filters with various Gaussian radius parameters of 1 mm, 3 mm, and 6 mm were deployed to produce filtered images for obtaining multi-scale texture features, from fine to coarse. The quantization of image grey levels was applied to normalize the image signal intensities. Grey-level intensities of the images were discretized with various settings of fixed bin counts, ranging from 50 to 350 with an incremental interval of 50. 

### 2.4. Feature Extraction

The extraction of radiomic features was performed using the publicly available PyRadiomics v2.2.0 and SimpleITK v1.2.4 package, which was embedded in the in-house developed Python-based v3.7.3 pipeline. Radiomic features were calculated from GTVn on CECT images, with and without LoG filters applied. They can be divided into three major families: shape, first-order statistics, and texture features, which can be further categorized into gray level difference matrix (GLDM), gray level cooccurrence matrix (GLCM), gray level run length matrix (GLRLM), gray level size zone matrix (GLSZM), and neighboring gray tone difference matrix (NGTDM) classes. In this study, a total of 2130 radiomic features, including 14 shape features, 72 first-order statistics, and 2044 texture features, were extracted from raw and LoG-filtered images under the pre-defined bin count settings. 

### 2.5. Model Development and Evaluation

The eligible patients from QEH were used for the development and internal validation of the prediction model, while those from QMH were used for an external independent evaluation of the trained model. For QEH, the eligible patients were randomly stratified into a training dataset and a validation dataset with 20 iterations, generating an ensemble feature set. For QMH, all the eligible patients were employed as an external testing set for assessing the model generalizability across medical centres. All the development and evaluation processes of the radiomic model were conducted by using R software v3.6.3.

**Figure 1:**
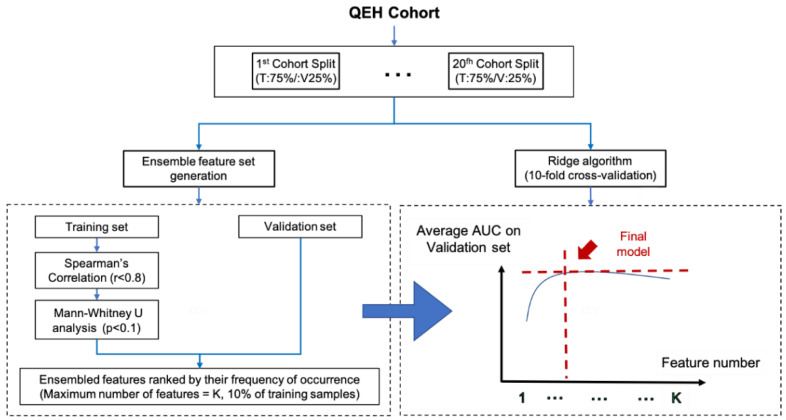
Workflow for radiomic model development.

For the radiomic (R) model, the entire QEH cohort was partitioned into a training set (~75%, *n* = 100) and an internal validation set (~25%, *n* = 35) with 20 iterations. In each iteration, z-score normalization was first applied to the training set to scale the value of each of the extracted features to a mean of 0 and standard deviation of 1, which was then applied to the internal validation set. In the training set, Spearman’s correlation (SC) analysis was performed using the “caret” package to assess inter-feature correlation; features which had an SC coefficient equal to or larger than 0.8 and had the greater mean absolute value between a pair of features were excluded. Unpaired two-sided Mann–Whitney U analysis was carried out using the “wilcox.test” function to examine the clinical association of each individual feature; features which showed a clinical association with a *p*-value of less than or equal to 0.1 were retained. Hence, a set of remnant features that had strong a clinical association to the prediction outcome and were free of highly redundant features was formed under each of the 20 iterations. A combination of these 20 feature sets formed an ensemble feature set, in which each feature was ranked according to its frequency of occurrence under the 20 iterations. Features with a higher frequency were retained for downstream model development. The maximum number of features in the final model was set to 10% of the training sample size [39,40,41]. In the case of exceeding the amount of features, features that had the least frequency of occurrence were excluded from the final R model. Ridge regression was employed for model development using the “glmnet” package in the R software. Meanwhile, a 10-fold cross validation was performed within the training set of each iteration to minimize the risk of model overfitting; nine of them were used for model training, followed by internal validation on the remaining partition. The final R model was selected when the model predictability reached its maximum on the validation cohort. A simplified workflow of the radiomic model development is illustrated in Figure 1.

For the clinical (C) model, a series of clinical data, including the patient’s gender, age, volume of GTVn, T-stage, N-stage, whether the patient had T-stage ≥ 3, N-stage ≥ 2, T-stage ≥ 3 plus N-stage ≥ 2, their pre-treatment body weight, pre-treatment body mass index (BMI), whether the patient had pre-treatment BMI ≤ 18.5, 18.5 < BMI < 22.9, 23 < BMI < 24.9, BMI > 23, and BMI > 25, were analyzed in the QEH cohort using an unpaired two-sided Mann–Whitney U test. Clinical data with a *p*-value of less than or equal to 0.01 were selected as the predictive features for the subsequent development of the C model. The finally selected radiomic features and clinical parameters were integrated for building the combined (RC) model.

The predictive performance of the models was evaluated using median area under receiver operating characteristic (AUC) curve using the “ROCR” package. Further, a bootstrap resampling technique with 1000 replications was applied to the entire QEH cohort for obtaining the 95%CI of the AUC estimates of the model. In each sub-sample, the model was trained using the selected features, resulting in an individual predictability in terms of the AUC. The results over all the 1000 replicates were then reported for the mean “corrected” AUC with a bias-corrected and accelerated 95%CI (BCa 95%CI). Furthermore, the developed models were independently evaluated on an external testing dataset of the QMH cohort for assessing the model generalizability across medical institutions. 

### 2.6. Statistical Analysis

The discriminability of the R model, in terms of the median AUC scores across the 20 iterations, was compared against the C and RC models in the training, validation, and testing datasets using Delong’s test. After bootstrapping, the “corrected” AUC and its BCa 95%CI were recorded and analyzed between the entire QEH and QMH cohort. On the other hand, a Chi-square test was employed to assess the statistical difference of the categorical patient clinical factors between the QEH and QMH cohorts, while an unpaired two-sided student *t*-test was applied for the continuous clinical factors. In all the above analyses, unless specified, a *p*-value of ≤ 0.05 was considered statistically significant.

## 3. Results

### 3.1. Patient Characteristics

A total of 124 and 58 NPC patients from QEH and QMH, respectively, were considered eligible in this study. There were 24 (~20%) and 16 (~28%) patients labelled as 1 in the QEH and QMH cohort, respectively. 

Table 1 summarizes the major characteristics of the patients in each studied cohort. It indicates that there was no statistically significant difference in the distribution of age, gender, histologic subtype, and volume of GTVn between the QEH and QMH cohort (*p* > 0.05), while there were significant differences in the distribution of a tumor’s T-/N-stage and the pre-treatment BMI of patients between the two cohorts (*p* < 0.05). However, further analyses of the clinical association of these features showed that neither the T-stage nor the N-stage of tumors were significantly associated with IfTMs in the QEH (*p* = 0.163 and *p* = 0.215, respectively) and QMH (*p* = 0.576, *p* = 0.443, respectively) cohort; pre-treatment BMI was found to be statistically significant in the QMH cohort (*p* = 0.014), but not in the QEH cohort (*p* = 0.600).

### 3.2. Model Development

**Figure 2:**
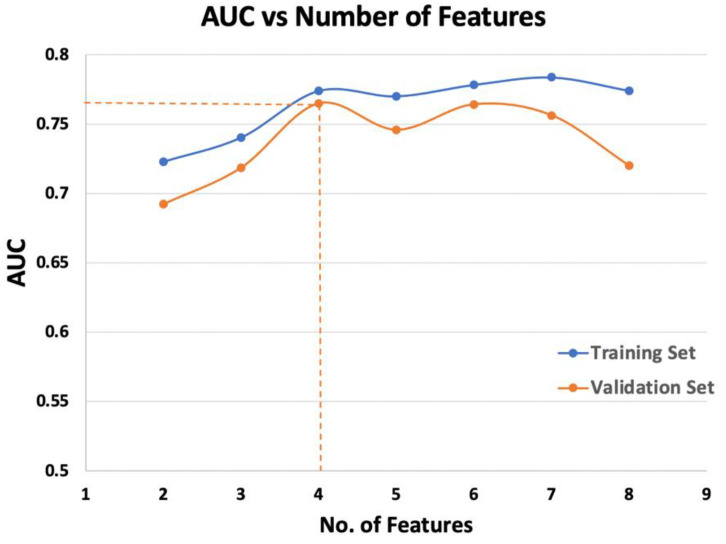
The change in AUC of the R model in both training and internal validation sets of the QEH cohort against the number of selected features.

Figure 2 illustrates the change in the AUC of the R model against the number of selected features in the model. The best-performing R model in the QEH internal validation set was determined when the number of selected features reached a value of 4, where the model predictability reached the maximum in the internal validation set. The selected features included LoG-6mm-glszm_Low Gray Level Zone Emphasis (Bin count = 100), LoG-6mm-glszm_Zone Entropy (Bin count = 50), Original_gldm_Large Dependence Low Gray Level Emphasis (Bin count = 300), and LoG-6mm_glcm_Inverse Variance (Bin count = 50).

For the C model, the results indicated that only N-stage ≥ 2 and the volume of GTVn were found to be significantly different between patients who experienced IfTM-triggered ART events and those did not in the QEH cohort (both *p* < 0.01). The four selected radiomic features and two clinical parameters were combined to form an ensemble of six features for the combined (RC) models.

### 3.3. Model Evaluation

Table 2 summarizes the performance of the different models (R, C, and RC) in the training and internal validation sets of the QEH cohort and the external testing set of the QMH cohort. The bootstrapped AUCs and the corresponding BCa 95%CI of the models in both the QEH and QMH cohorts were also calculated and reported.

From Table 2, it can be observed that the R model achieved the highest score of the “corrected” AUC at 0.784 (BCa 95%CI: 0.673, 0.859) in the QEH and 0.723 (BCa 95%CI: 0.534, 0.859) in the QMH cohort following the bootstrapping of 1000 replicates. The C model was the most under-performing model in both cohorts. Similarly, Delong’s test showed that the R model was significantly superior in predictability, in terms of the median AUC, over the C model in the training (0.753 vs. 0.624, *p* < 0.001), internal validation (0.716, 0.570, *p* < 0.01), and external testing (0.637 vs. 0.593, *p* < 0.001) sets. 

Apart from this, the addition of the two selected clinical features into the final R model (i.e., the RC model) did not yield better a predictive performance, with a “corrected” AUC of 0.782 (BCa 95%CI: 0.683, 0.862) in the QEH and 0.710 (BCa 95%CI: 0.474, 0.834) in the QMH cohort. Similarly, the external testing of the RC model demonstrated that there was no statistically significant difference in its predictability as compared to the R model (0.641 vs. 0.637, *p* = 0.816).

## 4. Discussion

NPC patients often present the involvement of neck LNs at presentation. Significant neck LN shrinkage is not uncommon in NPC patients undergoing RT, causing a risk of IfTMs during daily RT setup. If this occurs, tremendous efforts are made to implement ad hoc ART to ensure accurate and safe radiation delivery. However, the entire ART procedure for a single patient is highly time-consuming, resource-intensive, and requires multi-disciplinary efforts. Hence, the pre-treatment identification of individual patients who are at a greater risk of having an IfTM is highly desirable to alleviate the clinical workload, facilitate ART practice in the clinic, and achieve personalized RT. For the first time, we attempted to reveal the capability of CT-based neck nodal radiomics in predicting IfTM-triggered ART events in NPC patients via a multi-center setting in this study. 

The results of this study showed that radiomics plays a key role in predicting IfTM risk in NPC patients. The R model achieved the highest score of the “corrected” AUC at 0.784 (BCa 95%CI: 0.673, 0.859) in the QEH and 0.723 (BCa 95%CI: 0.534, 0.859) in the QMH cohort following the bootstrapping of 1000 replicates, achieving a profoundly superior predictability over the C model, which was found to be the most under-performing model in both cohorts (Table 2). Moreover, the combined RC model did not result in a better predictive performance than the R model, with the “corrected” AUC of 0.782 (BCa 95%CI: 0.683, 0.862) in the QEH and 0.710 (BCa 95%CI: 0.474, 0.834) in the QMH cohort; it also demonstrated no statistically significant difference in its predictability as compared to the R model in the external testing set (0.641 vs. 0.637, *p* = 0.816) (Table 2). To a degree, the superiority of radiomics may be ascribed to its unique property of unravelling tissue biologic characteristics in response to treatment perturbations. Indeed, an enormous number of articles in the literature have demonstrated the capability of radiomics in predicting tumor responsiveness on the basis of Response Evaluation Criteria in Solid Tumors (RECIST) [29,30,31,32,33,34,35,36], where the criteria are defined according to the degree of tumor volume shrinkage following treatment, which appears to follow the same line of thought as in this study. For example, Piao et al. investigated the potential of MR-based radiomics in differentiating NPC patients who are more likely to get a better treatment response from induction chemotherapy (IC) and those who are not; their radiomic model achieved an outstanding AUC of 0.905 [31]. Similarly, Wang et al. studied a diverse range of MR sequences for the prediction of early therapeutic response to IC in patients with esophageal carcinoma, yielding an AUC of 0.822 in their final model [30]. These studies have laid a great foundation for the radiomics prediction of neck tumor shrinkage leading to IfTM-triggered ART events in NPC patients, as in the present work.

Indeed, multiple research groups have reported a varying extent of neck lymph shrinkage during the course of RT treatment in head-and-neck cancer (HNC) patients, potentially triggering ART implementation. For example, Wing et al. quantified the anatomic changes of 30 NPC patients and reported that there was a significant regression of neck volumes over time with a mean loss rate of 0.39 ± 0.15%/day and a mean volume loss of 11.91 ± 5.57% upon treatment completion [22]. Murat et al. reported that there was a 43% reduction of the neck nodal target volumes in HNC patients undergoing RT [23]. Similarly, Cheng et al. reviewed both mid-treatment CT and MR scans of NPC patients and found out there were up to 30% reductions in the volume of nodal lesions [24]. All these investigations have suggested that the shrinkage of neck nodal lesions may serve as a favorable criterion for selecting patients for ART. However, there are limited studies on developing an ART eligibility screening strategy based on nodal tumor shrinkage. Yu et al. were the first to demonstrate the capability of MRI-based radiomics from primary tumors in predicting the ART eligibility of NPC patients. The performance of the prediction models in terms of AUC ranged from 0.75 to 0.93 in the testing datasets [13], which appears to be considerably higher than that in this study. To account for this, we inferred that the discrepancy may largely lie in the superiority of MRI in capturing tissue contrast over CT imaging. Given that the nodal lesions of NPC patients were mostly scanned with a CT imaging modality, instead of MRI, in the majority of the data available to us, the development of MR-based radiomic models was not feasible in this work. Nevertheless, investigations on the potential of MR-based nodal radiomics in predicting the ART eligibility of NPC patients can be an interesting area in the future.

Presently, the study conducted by Yu et al. is the only publication in the literature that attempted to predict the ART eligibility of NPC patients though MRI-radiomics from primary tumors (rather than CT-radiomics from metastatic lymph nodes as in this study) [13]. Radiomic features can be broadly divided into three categories: shape, first-order, and texture features. Notably, four texture radiomic features were included in the final R model in this study, including LoG-6mm-glszm-Low Gray Level Zone Emphasis, LoG-6mm-glszm-Zone Entropy, Original_gldm-Large Dependence Low Gray Level Emphasis, and LoG-6mm-glcm_Inverse Variance. On the contrary, the results from Yu et al. indicated that the majority of the selected features in the final radiomic models belonged to shape or first-order categories, with five out of eight in their contrast-enhanced T1-weighted model, and three out of six in both the T2-weighted and joint T1–T2 models [13]. Whether or not such a difference in the categorical distribution of the selected features between the two studies depends on the type of imaging modality (i.e., CT or MRI) or the source of features (i.e., primary NPC tumor or metastatic lymph nodes) or other factors remains to be fully elucidated; evidence from the body of literature on CT-based radiomics in predicting RECIST-defined treatment response (i.e., tumor shrinkage) may provide us with valuable insights for this. 

First, GLCM remains the common feature category in the final CT-based radiomic models, no matter if the source of the features was primary tumors or lymph node lesions [32,33,34,35,36]. Second, multiple studies on CT-based radiomics prediction for RECIST-defined treatment response share similar findings to this study. For instance, Coroller et al. analyzed CT-based radiomics from the primary tumor and lymph nodes of non-small cell lung cancer patients for predicting the pathological response, and reported that six out of the eight selected features from lymph nodes belonged to textural radiomic features [32]. Trebeschi et al. investigated the potential of CT-based radiomics from primary and lymph node lesions in predicting the cancer immunotherapy response, and found that 7 out of the 10 selected features were texture features [33]. Similarly, Santiago et al. assessed the nodal response of diffuse large B-cell lymphoma to treatment using CT-based radiomics, and reported that 6 out of the 10 selected features were texture features [34]. Moreover, Colen et al. developed a series of CT-based radiomic models for predicting the response to pembrolizumab in patients with advanced rare cancers, and all the selected features belonged to the texture category [35]. Third, the texture features of GLCM-Inverse Variance and GLSZM-Zone Entropy, selected in this current work, were also reported in previous studies on CT-based radiomics for treatment response prediction [33,34,35,36]. GLCM-Inverse Variance measures local homogeneity within the tissue volume and GLSZM-Zone Entropy measures texture irregularity or randomness quantified by the amount of homogeneous connected areas within the tissue volume of a certain size and intensity, describing the regional heterogeneity of the tissue [38]. Although intra-tumoral heterogeneity has been regarded to reflect tumor aggressiveness and hence its responsiveness (i.e., shrinkage) to treatment perturbations, the explicit correlation between these features and nodal tumor shrinkage remains unclear and deserves further exploration in the future. Based on findings from the above literature, it can be observed that the dominance of texture features in the R model of this study may be partly ascribed to the use of CT imaging and/or the feature source of lymph node lesions for treatment response prediction. Nevertheless, further investigations in this aspect are warranted.

This study has several limitations. First, the retrospective nature of this study might account for the potential bias; there were significant differences of the tumor T- and N-stage and the pre-treatment BMI of patients between the QEH and QMH cohort (Table 1). Nevertheless, these variables were in general not significantly associated with the IfTM-triggered ART events in both cohorts. Therefore, we speculated that its overall impact on the model development should be minimal. 

Second, the sample size involved in the model development and evaluation was relatively small, potentially causing model overfitting. To deal with this, a 10-fold cross-validation with 20 iterations and bootstrapping with 1000 replicates were applied in this work with an attempt to minimize the potential prediction bias. Moreover, an independent external test was performed to assess the model generalizability (i.e., degree of model overfitting) between medical centers. Nonetheless, a larger study cohort is warranted in the future to achieve a higher statistical inference. 

Third, the predictive performance of the radiomic model was limited by the use of CT images, which may impede its widespread clinical utility. However, this was limited by the dataset available to us, where the nodal lesions of NPC patients were mostly scanned with a CT imaging modality; thus, the development of MR-based radiomic models was not feasible in this work. 

Notably, the ART eligibility screening for NPC patients is still in its infant stage; this study is the first of its kind in investigating the potential of CT-based neck nodal radiomics in a multi-institutional setting for predicting IfTM-triggered ART demand in NPC patients. Hence, the findings of this study should provide valuable insights into developing a more effective screening for ART eligibility in NPC patients in the long run.

## 5. Conclusions

In this study, we attempted to investigate potential of CT-based neck nodal radiomics in a multi-institutional setting for predicting IfTM-triggered ART demand in NPC patients. The results of this work revealed that CT-based neck nodal radiomics was capable of predicting IfTM-triggered ART events in NPC patients undergoing RT. The R model consisted of four texture radiomic features, achieving a “corrected” AUC of 0.784 in the QEH cohort and 0.723 in the external QMH cohort. The findings of this study provide valuable insights for future study into developing an effective screening strategy for ART eligibility in NPC patients in the long run, ultimately alleviating the workload of clinical practitioners, streamlining ART procedural efficiency in clinics, and achieving personalized RT for NPC patients in the future. Future work on a larger cohort with MR nodal radiomics is highly warranted for strengthening the model predictability and statistical inference.

## Figures and Tables

**Table 1:** Distribution of patient characteristics in both QEH and QMH cohorts.

Patient Characteristics	QEH Cohort		QMH Cohort		*p*-Value
Age (average, range)	54.3 (27–81)		50.8 (32–81)		0.0591
Gender					0.329
Male (No.,%)	93	75	48	83	
Female (No.,%)	31	25	10	17	
WHO histologic subtype *					0.705
Type-1 (No., %)	3	2	1	2	
Type-2 (No., %)	2	2	2	3	
Type-3 (No., %)	119	96	55	95	
Tumor stage (7th AJCC)					
T-stage					<0.05
T1–T2 (No., %)	15	12	30	52	
T3–T4 (No., %)	109	88	28	48	
N-stage					<0.05
N0–1 (No., %)	19	15	23	40	
N2–3 (No., %)	105	85	35	60	
Pre-treatment BMI (average, range)	23.3 (14.3–35.5)		25.6 (17.9–35.8)		<0.05
Volume of GTVn (average, range)	30,025.7 (501.0–330,143.0)		22,808.6 (942.0–95,606.0)		0.233

* WHO histologic subtype of NPC: Type 1, keratinizing squamous cell carcinoma; Type 2, non-keratinizing differentiated carcinoma; Type 3, non-keratinizing undifferentiated carcinoma. Abbreviation: AJCC, American Joint Committee on Cancer.

**Table 2:** A summary of the performance of different studied models (R, C, and RC) in different studied cohorts.

Model	“Corrected” AUC (Average, BCa 95%CI)		Median AUC			*p*-Values		
	QEH Cohort	QMH Cohort	Training Cohort	Validation Cohort	Testing Cohort			
R	0.784 (0.673, 0.859)	0.723 (0.534, 0.859)	0.753	0.716	0.637	Reference		
C	0.648 (0.516, 0.747)	0.673 (0.499, 0.814)	0.624	0.570	0.593	**** ^1^	*** ^2^	**** ^1^
RC	0.782 (0.683, 0.862)	0.710 (0.474, 0.834)	0.757	0.679	0.641	0.488	**** ^1^	0.816

^1^ ****: *p*-value < 0.001; ^2^ *** 0.001< *p*-value < 0.01.

## Data Availability

The patients’ clinical and DICOM data are not publicly available for patient privacy protection purposes.
